# Secular trends in pregnancy weight gain in German women and their influences on foetal outcome: a hospital-based study

**DOI:** 10.1186/1471-2393-14-228

**Published:** 2014-07-15

**Authors:** Nina Ferrari, Peter Mallmann, Konrad Brockmeier, Heiko Klaus Strüder, Christine Graf

**Affiliations:** 1 Cologne Centre for Prevention in Childhood and Youth/ Heart Centre Cologne, University Hospital of Cologne, Kerpener Str. 62, 50937 Cologne, Germany; 2 Clinic and Polyclinic for Gynaecology and Obstetrics, University Hospital of Cologne, Kerpener Str. 34, 50931 Cologne, Germany; 3 Department of Paediatric Cardiology, Heart Centre Cologne, University Hospital of Cologne, Kerpener Str. 62, 50937 Cologne, Germany; 4 Institute of Movement and Neurosciences, Am Sportpark Müngersdorf 6, German Sport University Cologne, 50933 Cologne, Germany; 5 Department for physical activity in public health, Institute of Movement and Neurosciences, Am Sportpark Müngersdorf 6, German Sport University Cologne, 50933 Cologne, Germany

**Keywords:** Pregnancy, Gestational weight gain, Birth weight, Apgar, Obesity, Umbilical cord blood pH

## Abstract

**Background:**

Increasing rates of overweight have been reported. In Germany, women of childbearing age are especially affected. Those women are at increased risks of several peri- and postnatal complications. The purpose of this study was to carry out Germany’s first study in terms of secular trends of overweight and weight gain during pregnancy related to foetal clinical outcomes (birth weight, Apgar score and umbilical blood pH).

**Methods:**

A database maintained by a large regional university hospital in Cologne, Germany was used to evaluate clinical routine data from 1996 to 2012. 11771 women (23.5 ± 5.4 years; 18–48 years), who gave birth to a live singleton child (>2000 gram) were included. Recommended weight gain during pregnancy was based on IOM guidelines: Total weight gain range for underweight (initial BMI < 18.5 kg/m^2^) is 12.5 - 18 kg/ 28–40 lbs respectively, for normal-weight (initial BMI 18.5 -24.9 kg/m^2^) is 11.5 - 16 kg/ 25–35 lbs respectively, for overweight (initial BMI 25.0-29.9 kg/m^2^) is 7–11.5 kg/ 15–25 lbs respectively and for obese (initial BMI ≥ 30.0 kg/m^2^) is 5–9 kg/ 11–20 lbs respectively.

A one-way variance analysis was employed to test for differences in particular factors in various groups. Multiple linear regression analysis was used to model impact factors.

**Results:**

Over the second analysed period (2005–2012), the number of women with high weight gain increased from 33.8% to 42.9% (p <0.001). 54.5% overweight and 57.7% obese women were affected (p <0.001). Women with high weight gain were 54.5% significantly more likely to give birth to an infant ≥ 4000 grams than women with normal (31.7%) or low weight gain (13.8%, p < 0.001). Women with normal weight gain had significantly better foetal outcomes in terms of the Apgar score at 5 min and umbilical cord blood pH.

**Conclusion:**

These data confirm an increase in maternal weight gain before and during pregnancy. An excessive weight gain is accompanied by macrosomia, lower Apgar scores and pH-value. Women should therefore be advised about the risks of obesity before and during pregnancy as well as excessive maternal weight gain during pregnancy.

## Background

Findings from the largest nationally and internationally representative studies have shown that the number of overweight and obese adults is increasing [1-3]. Over the past two decades, in particular, the number of obese German women in younger age groups (up to 35) has increased [2]. Currently, 30% of 18 to 29 year old females show a BMI ≥ 25 kg/m^2^, 20.4% of these have been classified as pre-obese (25 to < 30 kg/m^2^) and 9.6% as obese (≥30 kg/m^2^). In the age group, 30–39 year olds, the prevalence reached 38% for overweight (BMI ≥ 25 kg/m^2^); 20.1% of these women have been classified as pre-obese and 17.9% as obese.

Evidence has been presented for links between overweight/obesity and a variety of co-morbidities during pregnancy. Overweight women seem to be twice as likely and obese women 3.3 times as likely to develop preeclampsia than normal-weight women (BMI < 25.0 kg/m^2^) [4]. Similarly, the risk of developing gestational diabetes is increased by 2.4% in overweight women and by 5.2% in obese women when compared to normal-weight women [4]. It is particularly noticeable that overweight and obese women gain much more weight during pregnancy than normal-weight women [5,6]. Excessive weight gain during pregnancy is associated with multiple maternal and neonatal complications like gestational hypertension, macrosomia, birth complications, caesarean delivery, stillbirth, low Apgar score at 5 minutes, hypoglycaemia or cardiovascular risk factors [7-11]. Women who gained more than the recommended amount of weight during pregnancy according to [12] were more likely to have offspring with greater BMI, waist, fat mass, lipid and inflammatory profiles [11]. In addition, studies have found excessive maternal weight gain [12] in overweight women to be associated with a higher percentage of fat mass in their children compared to children of normal-weight women [13]. Macrosomic new-borns are more at risk of developing metabolic disorders over the long term [14,15]. Adverse effects on foetal and neonatal parameters, described in terms of the Apgar score, are also more likely [16-21]. The children of overweight or obese women, for instance, more commonly achieve lower Apgar scores at five minutes than the children of normal-weight mothers [22,23]. Although the Apgar score is a reasonable parameter to evaluate the physical condition of new-born infants, it has some limitations. Therefore, the measurement of umbilical cord blood pH has been adopted as an addition to the Apgar score for assessing the condition of the new-borns [24]. A lower Apgar score is associated with lower levels of umbilical cord pH, especially in morbidly obese women (BMI >50 kg/m^2^) [25].

The objective of this analysis was therefore to carry out Germany’s first study into the secular trends of overweight, obesity and weight gain during pregnancy in relationship to foetal clinical outcomes (birth weight, Apgar, pH-value). In addition, it aimed to contribute to the improvement of very early prevention by identifying groups at risk for excessive weight gain and targeting them for preventive interventions.

## Methods

### Cohorts

Routine data from 18976 pregnant women registered at the Clinic for Gynaecology at the University Hospital of Cologne between 1996 and the summer of 2012 was retrospectively analysed by the Cologne Centre for Prevention in Childhood and Adolescence at the Heart Centre of the University Hospital of Cologne. Therefore, the study was exempt from ethical approval from the Ethics Committee of the University Hospital of Cologne.

Women who met the following criteria were included in the study’s analysis: women were 18–48 years of age when they gave birth, women possessed a minimum body height of one meter (3.28 feet), they were not experiencing a multiple pregnancy, and the child’s birth weight was at least 2000 grams. The study population was limited to children’s birth weight of at least 2000 grams, as there were many incomplete and/or implausible datasets in children weighing less than 2000 grams. Only data from 2000–2012 were used, because only this data had all exposure and outcome data available. Therefore, a total of 11771 pregnant women were included in the study. Metric measures are mainly presented in kilogram, gram and meter for better reading. Imperial measures are cited in tables as well.

Pre-pregnancy body weight as well as socioeconomic data were queried during the routine prenatal care visits in the Women’s Clinic. Anthropometric maternal data including age, height, pre-pregnancy weight, weight at the end of pregnancy and the resulting (relative) weight gain were assessed. BMI was calculated and classified according to the reference values issued by the World Health Organization (WHO); thus, a BMI ≥ 25 kg/m^2^ was considered overweight and ≥ 30 kg/m^2^ obese. The perinatal weight gain was defined as the difference between the weight before entering pregnancy and the weight determined just before delivery. The recommended weight gain during pregnancy was based on a function of the initial BMI (Table 1; [12]).

**Table 1:** **Weight gain recommendations during pregnancy according to the Institute of Medicine 2009**[12]

**Pre-pregnancy BMI**	**Total weight gain**	
	**Range in kg**	**Range in lbs**
Underweight (BMI < 18.5 kg/m^2^)	12.5 – 18	28 - 40
Normal-weight (BMI 18.5 – 24.9 kg/m^2^)	11.5 – 16	25 - 35
Overweight (BMI 25.0 – 29.9 kg/m^2^)	7 – 11.5	15 - 25
Obese (BMI > 30 kg/m^2^)	5 - 9	11 - 20

Source: Institute of Medicine (IOM) 2009; [12].

*kg =* kilogram; *lbs* = pounds.

Biometric data of the new-born (birth weight, umbilical blood pH, Apgar) were measured right after delivery. The Apgar scores at one and five minutes after birth serve as a surrogate parameter for the child’s health/the status of the new-born infant [26]. The Apgar score ranges from 0 to 10 and comprises a series of five individual tests performed on the new-borns: heart rate, respiratory effort, muscle tone, reflex irritability and colour. The umbilical artery blood pH at birth is commonly used to measure perinatal asphyxia and its measurement has been used as an adjunct to the Apgar score for assessing the new-borns condition [27].

This article considers the influence of pre-pregnancy BMI and maternal (relative) weight gain over the past 12 years on neonatal outcomes including birth weight (normal-weight 2000–3999 gram vs. ≥ 4000 gram), umbilical blood pH and (low) Apgar scores at one and five minutes after birth.

### Statistical analysis

The statistical analysis of the data set was performed using the SPSS 21.0 data-analysis software (Statistical Product and Service Solutions 21.0) for Windows™. Mean values and standard deviations (SD) were calculated using descriptive statistics for anthropometric data. The chi-square test was also conducted to determine the indirect association between two categorical variables. A one-way analysis of variance (ANOVA) was employed to test for differences in particular factors in various groups. A p-value less than 0.05 was considered to be significant. All confidence intervals (CIs) were estimated at the 95% level. Multiple linear regression analysis was used to model impact factors.

## Results

### Maternal parameters and general weight gain

Pre-pregnancy maternal anthropometric data is shown in Table 2.

**Table 2:** Pre-pregnancy maternal anthropometric data

	**N**	**Mean**	**SD**	**Range**
Maternal age	11771	32.5	5.4	18.0-48.1
Maternal height in cm (in)	11771	167.2 (65.8)	6.7 (26.4)	108.0-192.0 (42.5-75.6)
Maternal weight in kg (lbs)	11684	66.7 (147.0)	14.3 (31.5)	37.0-174.8 (81.6-385.4)
Maternal BMI^+^	11678	24.0	5.0	13.1-86.8
Maternal BMI classification (n;%)^$^: underweight (BMI < 18.5 kg/m^2^)	584 (5.0)			
Normal-weight (BMI 18.5 – 24.9 kg/m^2^)	7681 (65.8)			
Overweight (BMI 25.0 – 29.9 kg/m^2^)	2225 (19.1)			
Obese (BMI > 30 kg/m^2^)	1188 (10.2)			
Parity	11601	1.7	1.0	0-12

Pre-pregnancy maternal data of women who born a live singleton new-born between 2000 and 2012 at the Clinic for Gynaecology at the University Hospital of Cologne/Germany.

^+^Body Mass Index was calculated as bodyweight (kg) divided by height squared (m^2^); ^$^ Body Mass Index classification according to WHO guidelines*; cm =* centimetre; *in =* inch; *kg =* kilogram; *lbs* = pounds*; BMI =* Body Mass Index; *SD =* standard deviation; *Range* = minimum and maximum.

5.0% of the women (n = 584) were considered underweight, 65.8% (n = 7681) normal-weight, 19.1% (n = 2225) overweight and 10.2% (n = 1188) obese before pregnancy. Table 3 shows the development of weight gain while taking the prenatal weight classification into account.

**Table 3:** Total weight gain during pregnancy (mean) and 95% confidence intervals according to the pre-pregnancy body mass index

**BMI classification**^**+**^	**n**	**Mean** ± **SD in kg**	**Mean** ± **SD in lbs**	**95% Cl in kg (lbs)**
underweight (BMI < 18.5 kg/m^2^)	478	13.6 ± 5.0	30.0 ± 11.0	13.1-14.1 (28.9-31.1)
normal-weight (BMI 18.5 – 24.9 kg/m^2^)	6058	13.8 ± 5.2	30.4 ± 11.5	13.7-13.9 (30.2-30.6)
overweight (BMI 25.0 – 29.9 kg/m^2^)	1740	12.6 ± 5.9	27.8 ± 13.0	12.3-12.8 (27.1-28.2)
obese (BMI > 30 kg/m^2^)	917	10.7 ± 6.9	23.6 ± 15.2	10.3-11.0 (22.7-24.3)

^+^Body Mass Index classification according to WHO guidelines; *BMI* = Body Mass Index; *SD =* standard deviation; *kg* = kilogram; *lbs* = pounds; *95% Cl:* 95% confidence intervals in kilogram and (pound).

Gestational weight gain averaged 13.3 ± 5.6 kg and, when the different years were taken into account, it became evident that women gained less weight in 2000 (12.3 kg; 95% Cl: 11.9, 12.7), in 2001 (12.3 kg; 95% Cl: 11.9, 12.6) and in 2002 (12.5 kg; 95% Cl: 12.0, 12.9) than women in 2010 (13.7 kg; 95%Cl: 13.4, 14.1), 2011 (13.9 kg; 95% Cl: 13.5, 14.2) and 2012 (13.7 kg; 95% Cl: 13.2, 14.2) (p <0.05).

### Relative weight gain

**Figure 1:**
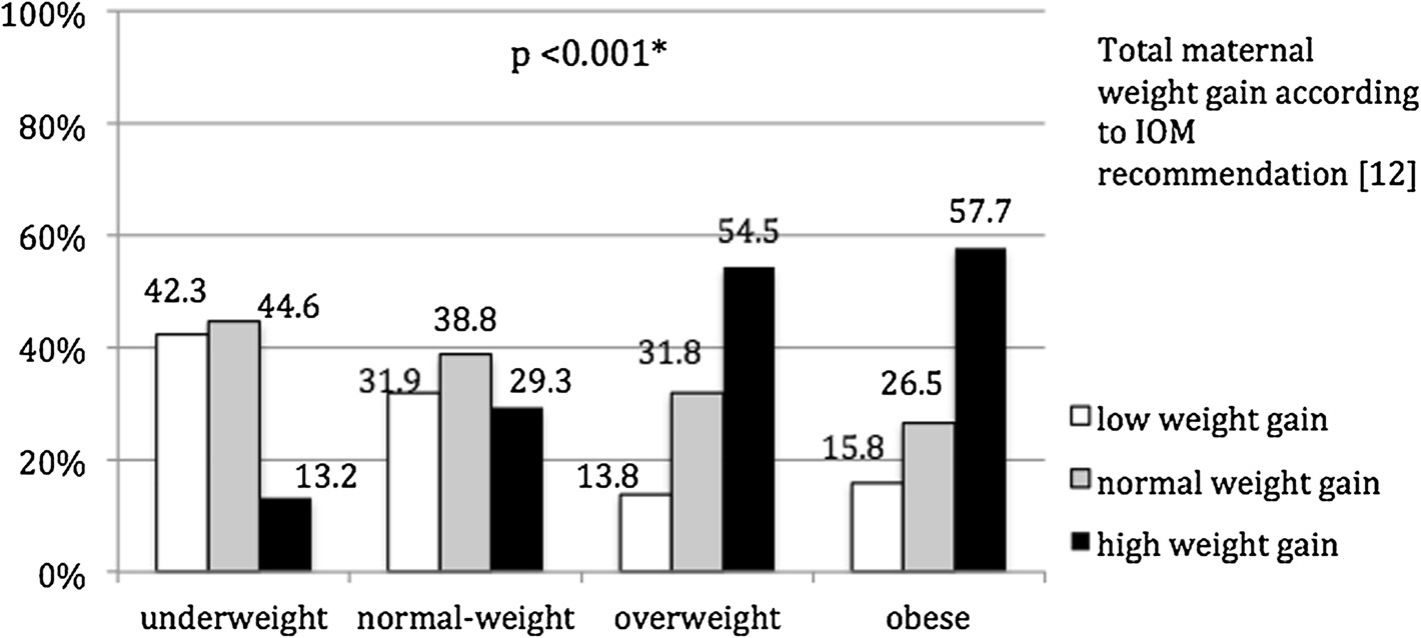
**Weight gain according to IOM guidelines [**[12]**] depending on weight classification.** *Chi^2^-test.

**Figure 2:**
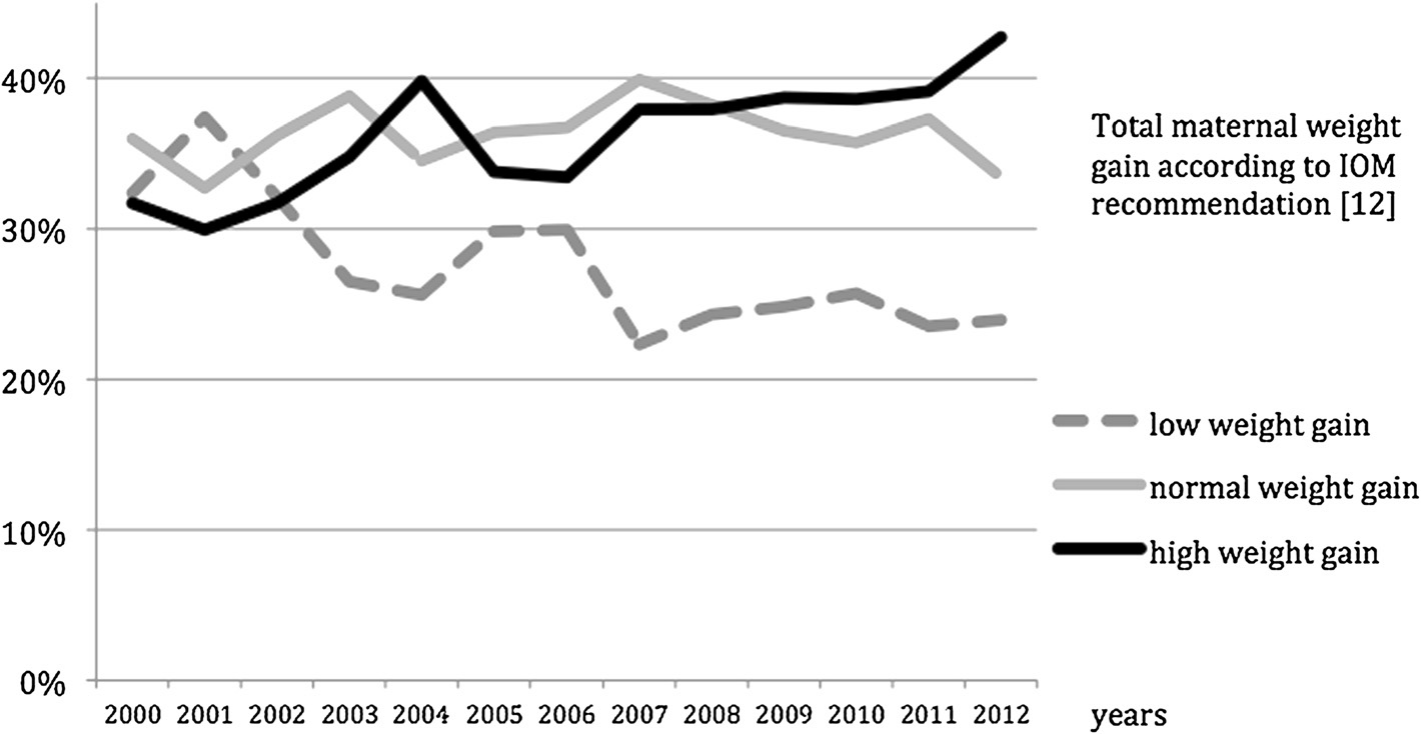
**Total weight gain during pregnancy according to IOM guidelines [**[12]**] depending on the year.** *Chi^2^-test.

Depending on weight before pregnancy [12], 27.4% of the women (n = 2514) gained less weight than the recommended amounts, 36.5% (n = 3347) were within the range and 36.0% (n = 3303) gained more than the recommended amount (p <0.001; see Figure 1). 54.5% of the overweight and 57.7% of the obese pregnant women gained more than the recommended amount, than underweight and normal-weight women (13.2% and 29.3% respectively).From 2000 to 2012, the values show a trend towards excessive weight gain (p <0.001). Especially over the second period (2005 to 2012), the number of women who experienced excessive gestational weight gain increased markedly from 33.8% to 42.9% (Figure 2).

### Birth weight

**Figure 3:**
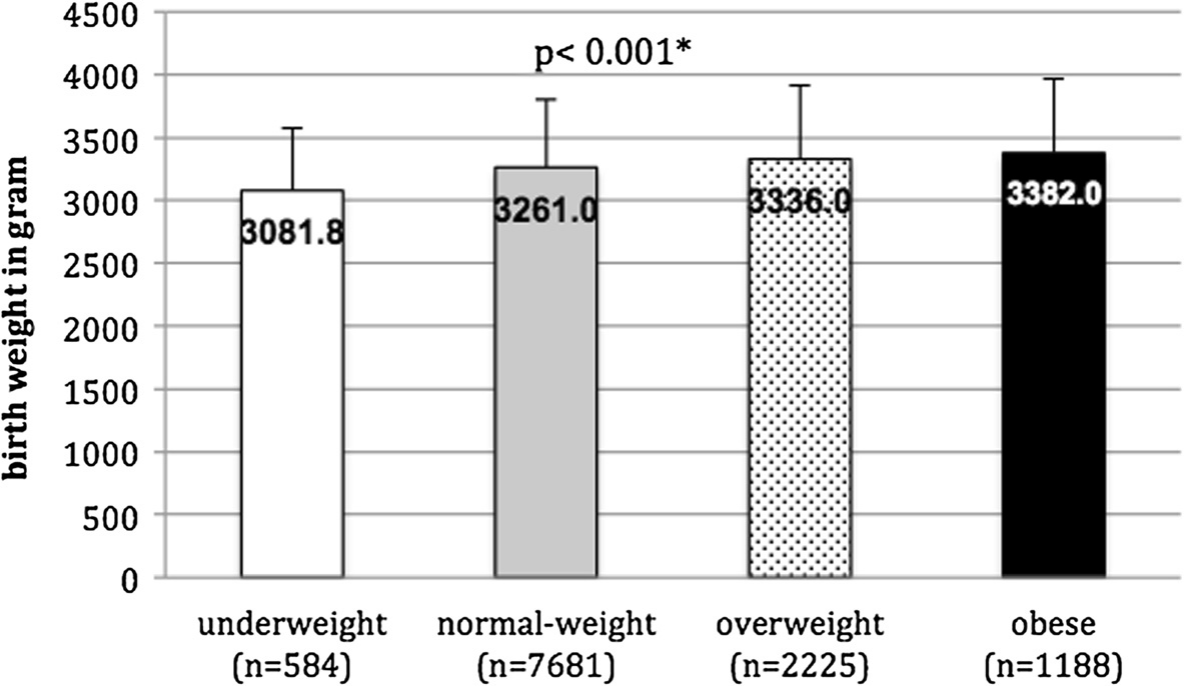
**Birth weight in gram by pre-pregnancy weight classification.** *one-way ANOVA.

The new-borns’ weight at birth averaged 3280.9 ± 552.4 g. No significant differences in birth weight were observed during the period from 2000 to 2012.On average, the children born to underweight women weighed 3081.8 g (95% Cl: 3037.2, 3126.4), those born to normal-weight women weighed 3261.0 g (95% Cl: 3248.8, 3273.0), those born to overweight women weighed 3336.0 g (95% Cl: 3313.2, 3358.9) and those born to obese women weighed 3382.0 g (95% Cl: 3350.0, 3413.3). A significant difference between all groups was found (p <0.05) (Figure 3).

### Birth weight depending on relative weight gain

At 3412.4 ± 552.8 grams, infants were significantly heavier from women who had higher weight gain than those who had normal or low weight gain during pregnancy (each p <0.001; see Table 4). This finding was confirmed when birth weights ≥ 4000 grams were examined. Women who gained more than the recommended weight were significantly more likely to give birth to an infant ≥ 4000 g (54.5%, n = 458) than women who were within (31.7%, n = 267) or below the recommendations (13.8% n = 116, p <0.001).

**Table 4:** Mean and 95% confidence intervals (CIs) for birth weight in gram by total maternal weight gain

**Total maternal weight gain**	**n**	**Mean**	**SD**	**95% Cl**
**Low weight gain**	2514	3107.7	527.4	3086.5 - 3128.9
**Normal weight gain**	3347	3258.2	541.0	3239.8 – 3276.5
**High weight gain**	3303	3412.4	552.8	3394.0 – 3430.9

^+^The recommended weight gain during pregnancy was based on a function of the initial BMI according to IOM [12]; *SD* = standard deviation; *95% Cl:* 95% confidence intervals.

### Regression analysis

Multiple linear regression analysis was carried out to analyse the individual factors that had an impact on the children’s weight at birth. The initial model included parity, country of origin (Germany or others), maternal age, pre-pregnancy weight, weight gain, marital status (single, married, widowed, divorced) as well as the mother’s employment status (employed or unemployed). In the final model, the variables of weight gain (β-coefficient: 0.228; p <0.001), parity (β-coefficient: 0.092; p <0.001), maternal age (β-coefficient: 0.029; p = 0.008), pre-pregnancy weight (β-coefficient: 0.169; p <0.001), mother’s employment (β-coefficient: −0.063; p <0.001), marital status (β-coefficient: 0.032; p = 0.002), and country of origin (β-coefficient: 0.027; p = 0.010) explained 8.4% of the variance.

### Foetal parameters

**Figure 4:**
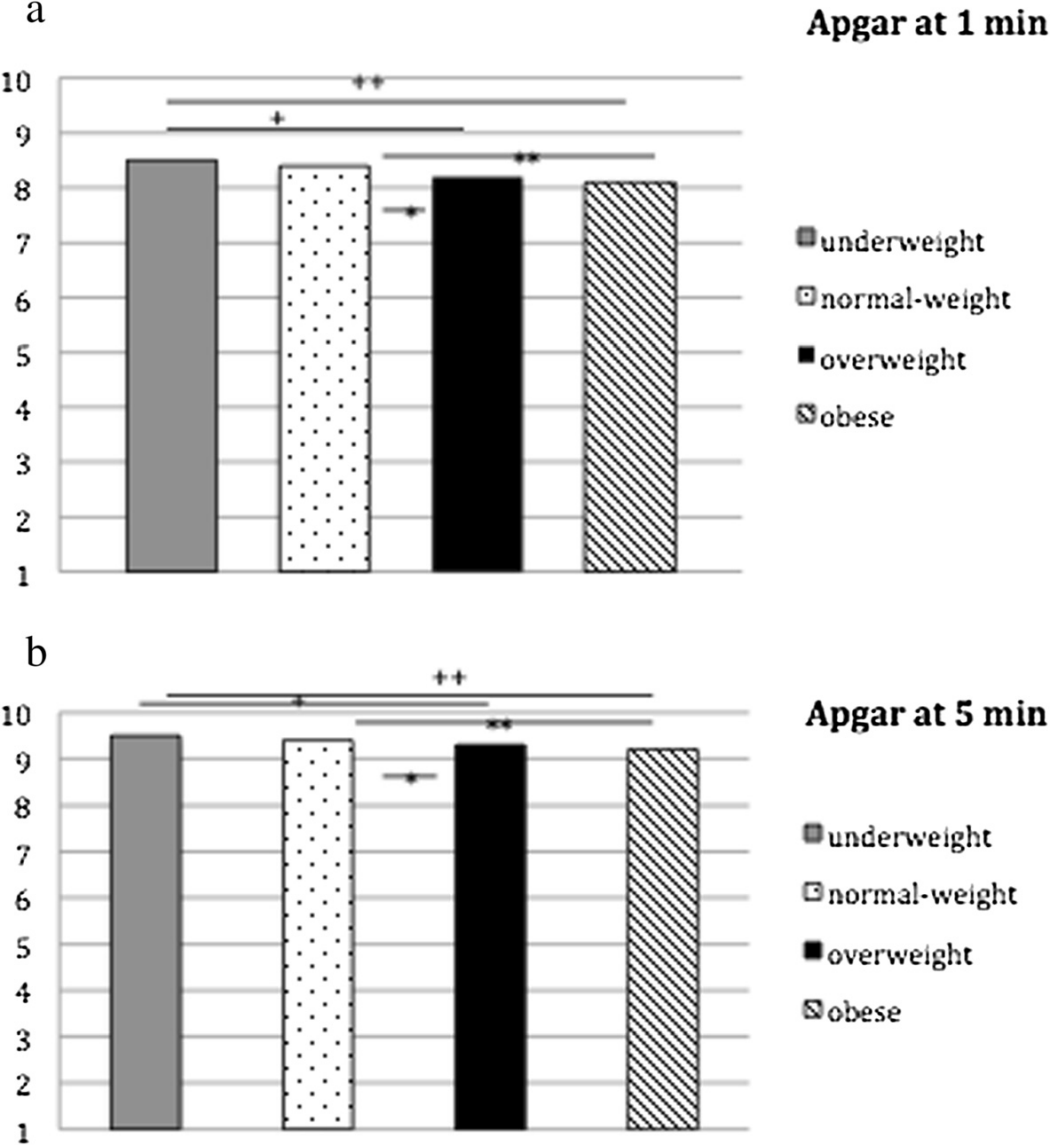
**Apgar score at 1 and 5 minutes depending on weight classification. a**. Apgar score at 1 min: depending on weight classification; One way Anova p <0.001; ^+^Apgar score at 1 min between underweight vs overweight women p = 0.014; ^++^Apgar score at 1 min between underweight vs. obese women p <0.001; ^*^Apgar score at 1 min between normal-weight vs overweight women p = 0.003; ^**^Apgar score at 1 min between normal-weight vs. obese women p <0.001. **b**. Apgar score at 5 min; One way Anova p <0.001 ^+^Apgar score at 5 min between underweight vs overweight women p = 0.014; ^++^Apgar score at 5 min between underweight vs. obese women p = 0.001; ^*^Apgar score at 5 min between normal-weight vs overweight women p = 0.020; ^**^Apgar score at 5 min between normal-weight vs. obese women p <0.001.

The average Apgar index at one minute after birth was 8.3 ± 1.7 and 9.4 ± 1.3 after five minutes. The average umbilical blood pH-value was 7.3 ± 0.2. There was a significant correlation between Apgar score at one and five minutes and the pH-value (r = 0.085, p <0.001; r = 0.065, p <0.001). No differences were found between pH-value and BMI-classification, whereas there was a significant difference in weight classes to Apgar values at one and five minutes after birth between all groups (each p <0.001). The new-born infants of obese and overweight women showed significantly lower Apgar values at one (p <0.001; p = 0.003) and at five minutes (p <0.001; p = 0.020) compared with the children of normal-weight women (Figure 4).

There was a significant difference between recommended weight gain during pregnancy and Apgar score at 5 min (p = 0.013 adjusted by mode of delivery and umbilical cord blood pH) as well as recommended weight gain during pregnancy and umbilical cord blood pH (p = 0.036 adjusted by mode of delivery and Apgar score at 5 min).

Multiple linear regression analysis was used to identify possible impact factors on the Apgar score at five minutes. Birth weight, marital status, pre-pregnancy weight, mother’s age, gestational weight gain during pregnancy, parity, umbilical blood pH and mode of delivery were included in the model. In the final model, the variables of birth weight (β-coefficient: 0.224; p <0.001), pre-pregnancy weight (β-coefficient: −0.075; p <0.001), mother’s age (β-coefficient: 0.023; p = 0.031), parity (β-coefficient: −0.036; p = 0.001), pH value (β-coefficient: −0.067; p <0.001), delivery mode vaginal operative (β-coefficient: 0.099; p <0.001), delivery mode elective caesarean section (β-coefficient: 0.087; p <0.001) and delivery mode emergency caesarean section explained 9.0% of the variance.

## Discussion

Excess weight and obesity during pregnancy result in major adverse effects on both mother and child later in life [19,28,29]. The Avon Longitudinal Study of Parents and Children (ALSPAC), for instance, revealed that children born to women who experienced excessive gestational weight gain will be at risk of becoming obese and suffering elevated blood pressure as well as elevated blood parameters significantly more often than their normal weight gain counterparts [11]. The weight gain during pregnancy was correlated to the baseline BMI of the mothers [6,9]. Many findings to date have described possible long-term effects [30,31] but there are relatively few studies into such short-term effects as congenital malformations and mortality. The five-minute Apgar score is used as a potential surrogate parameter for physical condition, even if it has some limitations [26]. Experts recommend combining the Apgar score with other more objective neonatal outcomes like umbilical cord blood pH. No data related to secular trends in Germany have yet been published. The presented study, therefore, investigated the data from a regional database for the Cologne area to establish (relative) weight gain during pregnancy and possible associations with foetal parameters (birth weight, Apgar score and umbilical blood pH). Especially over the second analysed period (2005 to 2012), the number of women who experienced excessive gestational weight gain increased markedly from 33.8% to 42.9%. Predominantly the overweight and obese women gained more weight than recommended. DeVader et al. [32] indicated that women experiencing excessive weight gain during pregnancy but who were classified as being of normal weight prior to conception and before putting on too much weight, were at higher risk of preeclampsia, were more likely to require induced labour and caesarean delivery, and had a higher ratio of infants who were large for their gestational age.

In our study, multiple linear regression analysis showed that weight gain during pregnancy had the highest impact on birth weight, although the total variance explained only 8.4%. We were able to assess and adjust our results for confounding bias. We acknowledge that our results may be influenced by other important confounders, including gestational age or dietary habits of the women in our study population.

Nevertheless, in our study, foetal outcomes attributed to high maternal weight gains are reflected in increased birth weight, lower Apgar scores at one and five minutes after birth, and lower umbilical cord blood pH compared with women who gained weight within the recommended range. Consistent with our study, Stotland et al. [9] also provided evidence that underlined a relationship between excessive weight gain, large size for gestational age, lower Apgar scores (<7) at five minutes, and lower umbilical cord blood pH. This may influence the development of infants. Macrosomic new-borns are more likely than other infants to be obese in childhood, adolescents and early adulthood [33] and are at risk of cardiovascular and metabolic risk factors later in life [15,34]. In addition, low Apgar scores and lower umbilical cord blood pH are associated with neonatal mortality and morbidity, spasticity and long term outcomes like cerebral palsy [27,35,36]. Our data showed, that birth weight had the highest impact on Apgar score at five minutes, although the total variance is very small and findings must be interpreted with caution. Nevertheless, our findings demonstrate that prevention of maternal excessive weight gain is important.

It should be noted that the presented study is limited to the extent that it only recorded anthropometric data before pregnancy; smoking was not recorded as well as gestational age, and that it only considered a regional situation. It was possible to adjust our results for some confounding bias, but not all. Although studies have often reported outcomes in terms of birth weight, it should be noted that gestational age has an important impact on foetal parameters. Gestational age is strongly associated with birth weight [37]. In addition, the Apgar score at one and five minutes after birth are directly related to gestational age [38]. Nevertheless, Kitlinski et al. 2003 [39] found that within the interval of 37–41 weeks no association between gestational age and a low Apgar score was demonstrated, but a statistically significant positive association between a gestational age of 41 3/7 weeks or more and Apgar score less than 7 at 5 minutes was found. Furthermore, the authors demonstrated that the mean umbilical artery pH decreased with increasing gestational age. Therefore, a substantial limitation of our retrospective study might be that data of gestational age was not available. Another limitation might be the Apgar score. The Apgar score, as a relatively subjective parameter, is an expression of the infants’ physiological condition. Therefore, we used birth weight and umbilical blood pH as well, to define foetal outcome and neonatal condition [9,24,27,40].

## Conclusion

In conclusion, the presented study demonstrated that the prevalence of excessive weight gain during pregnancy has increased markedly in the Cologne area over recent years and that these results are closely associated with birth weight, low Apgar score and umbilical blood pH. The main strengths of the study are its consistent method, dataset over 12 years, and the size of the examined collective.

Despite the mentioned limitations, our results confirm the importance and contribute to the improvement of early preventive measures by identifying women at risk for excessive weight gain. The prevention of maternal excessive weight gain is likely to benefit women’s health as well as that of their infants. Women should therefore be advised about the risks of obesity during pregnancy prior to conception and should be motivated by gynaecologists and midwives to take initial steps to avoid excessive weight gain during pregnancy.

## Abbreviations

ANOVA: Analysis of variance; BMI: Body mass index; HDL: High density lipid; i.e.: In example; in: Inch; IOM: Institute of Medicine; Lbs: Pound; SD: Standard deviation; WHO: World Health Organization.

## Competing interests

The authors declared that they have no conflict of interests.

## Authors’ contribution

Study concept: NF, PM, KB, CG; Data collection: PM; Data handling: NF, PM, CG; Analysis: NF; Interpretation of data: NF, CG; Critical revision of the manuscript: PM, KB, HKS, CG. All authors read and approved the final manuscript.

## Pre-publication history

The pre-publication history for this paper can be accessed here:

http://www.biomedcentral.com/1471-2393/14/228/prepub
